# Serial passage in an insect host indicates genetic stability of the human probiotic *Escherichia coli* Nissle 1917

**DOI:** 10.1093/emph/eoac001

**Published:** 2022-02-11

**Authors:** Nicolas C H Schröder, Ana Korša, Haleluya Wami, Olena Mantel, Ulrich Dobrindt, Joachim Kurtz

**Affiliations:** 1 Institute for Evolution and Biodiversity, University of Münster, Münster, Germany; 2 Institute for Hygiene, UKM Münster, Münster, Germany

**Keywords:** probiotics, serial passage, invertebrate host, *E. coli*

## Abstract

**Background and objectives:**

The probiotic *Escherichia coli* strain Nissle 1917 (EcN) has been shown to effectively prevent and alleviate intestinal diseases. Despite the widespread medical application of EcN, we still lack basic knowledge about persistence and evolution of EcN outside the human body. Such knowledge is important also for public health aspects, as in contrast to abiotic therapeutics, probiotics are living organisms that have the potential to evolve. This study made use of experimental evolution of EcN in an insect host, the red flour beetle *Tribolium castaneum,* and its flour environment.

**Methodology:**

Using a serial passage approach, we orally introduced EcN to larvae of *T.castaneum* as a new host, and also propagated it in the flour environment. After eight propagation cycles, we analyzed phenotypic attributes of the passaged replicate EcN lines, their effects on the host in the context of immunity and infection with the entomopathogen *Bacillus thuringiensis*, and potential genomic changes using WGS of three of the evolved lines.

**Results:**

We observed weak phenotypic differences between the ancestral EcN and both, beetle and flour passaged EcN lines, in motility and growth at 30°C, but neither any genetic changes, nor the expected increased persistence of the beetle-passaged lines. One of these lines displayed distinct morphological and physiological characteristics.

**Conclusions and implications:**

Our findings suggest that EcN remains rather stable during serial passage in an insect. Weak phenotypic changes in growth and motility combined with a lack of genetic changes indicate a certain degree of phenotypic plasticity of EcN.

**Lay Summary:**

For studying adaptation of the human probiotic *Escherichia coli* strain Nissle 1917, we introduced it to a novel insect host system and its environment using a serial passage approach. After passage, we observed weak phenotypic changes in growth and motility but no mutations or changes in persistence inside the host.

## INTRODUCTION

Probiotics are the most prominent defensive microbes in humans. They are in use for thousands of years and studied for over a hundred years as therapeutics for dermal, vaginal and intestinal infections [1–3]. Oral intake as a treatment against various gastrointestinal disorders, including severe bacterial infections, is the most widespread application of probiotics [4]. As antibiotic resistance of pathogens has already developed into a global public health problem with unforeseeable severity, the need for safe and well-characterized biotherapeutics asks for further research on the protective mechanisms of established probiotics [5, 6].

A commonly used and medically important probiotic is the gram-negative bacterium *Escherichia coli* Nissle 1917 (EcN). It belongs to the large family of *Enterobacteriaceae* and the *E.coli* serotype O6:K5:H1 [7]. EcN was introduced as the pharmaceutical preparation Mutaflor^®^ by Alfred Nissle, after isolating it from the feces of a WWI soldier and characterizing its antagonistic effects against pathogenic *Enterobacterales* [8]. To date, Mutaflor^®^ (Pharma-Zentrale GmbH, Germany) is a commercially available drug against intestinal diseases like Crohn’s disease and ulcerative colitis .

While the microbial characteristics [9] and the genetic background [10, 11] of EcN are well-studied, the mechanisms of its probiotic activity remain elusive. The protective effects of EcN are considered to be linked to several particular properties and fitness factors. EcN secretes two bactericidal microcins, which have been shown to drive microbial competition [12, 13]. Biofilm formation as well as outcompeting pathogens regarding iron uptake [14] are thought to be factors contributing to the probiotic properties of EcN [15].

EcN has shown high colonization success in gnotobiotic rats [16] and piglets [17] upon oral exposure, while also most conventionally kept mice and rats retain a long-term EcN colonization of their guts [18]. In humans, however, only certain specific conditions allow stable, long-term colonization of the intestines. An extensive clinical study on healthy adults suggests that a natural intestinal microbiome might prevent a long-term colonization by EcN and that the kinetics of EcN intestinal colonization in the human system are highly variable [19].

In addition to the importance for colonization, the communication of EcN with epithelial cells has been shown to have numerous effects on the host’s immune system [20–22]. Well-studied immunomodulatory effects are the induction of the antimicrobial peptide (AMP) human β-defensin-2 (HBD2) [23] and the regulation of T-cell activation, expansion and apoptosis [20, 24, 25]. Directly interacting with the intestinal epithelial cells, EcN is proposed to prevent a compromised epithelial barrier, which is considered a key mechanism in the development of intestinal diseases [26]. EcN has been shown to inhibit “leaky gut” symptoms, which are associated with diseases like coeliac or Crohn’s disease, by enhancing the integrity of the mucosal surface of the intestinal epithelium [26–28].

Although application of EcN is usually safe, a few studies call for careful usage under specific conditions [29–32]. As a safety-relevant aspect, not much is yet known about the phenotypic and genomic plasticity of EcN under different growth conditions that may contribute to bacterial adaptation to different hosts or environments. In contrast to abiotic therapeutics, probiotics are living organism that have the potential to evolve, both in the mammalian gut and in the environment. However, even though EcN is so widely used, studies of its evolution are still scarce. Crook *et al.* [33] recently demonstrated in-host evolution of EcN in the mouse gastrointestinal tract over several weeks. Evolution was dependent on diet and background microbiota, which may have implications regarding the large variability observed for the probiotic efficacy [34]. A deepened understanding of the evolution of EcN under diverse conditions will allow for more accurate prediction of potential phenotypic or genetic changes that may happen when EcN is used as a probiotic and it may thus also have implications for human health issues.

While mammals remain the most widely used model systems in research on infectious diseases and host–microbe interactions, alternative models might provide important benefits and are increasingly recognized as promising alternatives [35, 36]. Among other factors, high costs, time-consuming maintenance and the ethical concerns of infecting mammals with pathogens drive the substitution of insect models for mammals as host systems [37–39]. Regarding microbial pathogenesis, insects and mammals show certain parallels in the structure of protective tissues (reviewed in Ref. [37]) and they share a highly conserved innate immune system with analogous signaling pathways [40, 41].

*Tribolium castaneum* is an established model organism and has all the benefits of insect models: a short life cycle, high fecundity and inexpensive maintenance, allowing experiments with large sample sizes [42]. It allows for RNAi studies using specific and efficient knockdowns of genes by larval or parental dsRNA injection [43, 44]. As a member of the largest eukaryotic order, Coleoptera, *T.castaneum* makes a more suitable representative of other insects, since it is evolutionary more basal than Lepidoptera and Diptera [45]. That makes the red flour beetle a commonly used insect model in various fields of research including development, evolution, immunity and host–pathogen interactions [46–49]. Moreover, identification of various extra- and intracellular signaling pathways of the innate immune system, including many proteins with human homologs [50] allows for extensive and conclusive studies of the *T.castaneum* immune system and its interaction with microbes. *Triboliumcastaneum* and its pathogens have become well-established models, in particular for immuno-ecological and evolutionary studies [48, 51–56]. Additionally, *T.castaneum* has been proposed as a screening system for potential drugs as well as pharmaceutical side effects [57, 58]. Grau *et al.* [59] used *T.castaneum* for *in vivo* characterization of a probiotic *Enterococcus mundtii* isolate from *Ephestia kuehniella* larvae and suggested *T.castaneum* as an alternative model for the pre-screening of probiotics.

We here aimed to make use of these strengths of the *T.castaneum* system to further investigate the possible adaptation of EcN to a new host and its environment. To better understand phenotypic and genomic plasticity of EcN as mechanisms that may contribute to bacterial host adaptation, a serial passage experiment was performed, using eight cycles of intestinal colonization in beetle larvae and their flour environment (Supplementary Fig. S1). We also assessed the effects of an EcN treatment on the host by monitoring life history traits (survival, pupation, eclosure) and measuring expression levels of AMPs in *T.castaneum* larvae after oral exposure. Additionally, a putative protective effect against the beetlés natural pathogen *Bacillus thuringiensis tenebrionis* (*Btt*) was analyzed by coinfecting larvae with EcN and *Btt.* We observed only weak phenotypic differences of both, beetle- and flour-passaged EcN in motility, growth and colony morphology, compared to the ancestral EcN. This suggests that EcN had adapted to these novel environments. However, slightly changed conditions revealed that the phenotypic changes might not be stable. Moreover, we did not observe any genetic changes, suggesting that adaptation of EcN had happened on the phenotypic and/or epigenetic level, while this important probiotic remains genetically stable.

Even though we did not observe any effect on the host, changes in phenotypic attributes suggest that EcN could phenotypically adapt to novel environments or unusual hosts, such as during the intestinal passage in *T.castaneum* larvae.

## METHODOLOGY

### Model organisms

#### Tribolium castaneum

All experiments in this work involving red flour beetles were conducted with the *T.castaneum* strain Cro1, which was collected from a granary in Croatia in Summer 2010 [48]. The beetles were kept on heat sterilized (75°C; overnight) all-purpose wheat flour (Type 550) with 5 wt.% yeast at 30°C and 70% air humidity. The incubators were set up for a 12 h/12 h light-dark cycle. Synchronization of age was achieved by transferring ∼1-month-old adult beetles to a cultivation box containing fresh flour with yeast. After 24 h of oviposition, the adults were removed by sieving the flour with a metal sieve with a mesh size of 720 µm (Retsch).

#### *Escherichia coli* Nissle 1917

The *Escherichia coli* Nissle 1917 (EcN) strain we used for the serial passage experiment was obtained from Ardeypharm GmbH (Herdecke) as the probiotic product Mutaflor^®^. For long-term storage, glycerol was added to an LB (lysogeny broth) overnight culture (25% final glycerol concentration). It was stored in 500 µl microtubes as 200 µl aliquots of 100 µl at −80°C. Single colonies for lab use of EcN were generated by streaking out the −80°C glycerol culture on LB agar plates and incubating at 30°C overnight. Adding a 40 µg/ml Congo Red and 20 µg/ml Coomassie Brilliant Blue, the LB agar medium produces red agar plates that are used for the characterization of EcN colonies. EcN forms characteristic red dry and rough colonies on this agar, by incorporating the dyes in the colonies’ biofilm. This mechanism is used to selectively identify EcN colonies.

#### *Bacillus thuringiensis* bv. *tenebrionis*

The *Bacillus thuringiensis* bv. *tenebrionis* (*Btt* [60]) strain used in this work was acquired from the *Bacillus* Genetic Stock Center of the Ohio State University (USA) and was stored in 500-µl microtubes as 100-µl aliquots with 25 vol.% glycerol. Single *Btt* colonies were generated by streaking out the −80°C glycerol culture on LB agar plates and incubating at 30°C overnight.

### Serial passages

#### Experimental design

A central aim of the serial passage experiment was to establish EcN lines that are able to stably colonize the *T.castaneum* host. For the ancestral EcN, preliminary experiments showed an exposure-time-dependent EcN persistence of 4–7 days in *T.castaneum* larvae, upon oral exposure (Supplementary Fig. S1). Eight oral uptake cycles were performed by introducing EcN to the environment of the larval midgut using oral exposure.

Originating from one ancestral clone, 10 EcN replicate lines were passaged (L1–10) in the host (larvae-passaged), while six replicate lines served as controls for the larval environment (flour-passaged) without being exposed to *T.castaneum* (Supplementary Fig. S2).

#### Liquid cultures

Initially, frozen ancestral EcN stock cultures and later EcN cells extracted from larvae were plated on LB agar plates dyed with Congo Red and Coomassie Blue and incubated overnight at 30°C. The following day, 50 ml of LB were inoculated with a single colony and incubated at 30°C and 180 rpm overnight. After centrifugation and washing the cultures with phosphate-buffered saline (PBS) at 4000 g at room temperature (RT) for 10 min, the cell concentrations were standardized to 8 × 10^9^ cells/ml using an approximation based on the optical density (turbidity) of the sample, which was measured at a wavelength of 600 nm (OD_600_). The OD_600_-based calculations were calibrated using an EcN-specific standard curve (produced previously, data not shown), which was verified by streaking out several dilutions of measured samples and counting colony-forming units. Aliquots of cell suspensions with adjusted concentrations were stored as frozen glycerol cultures for each line and passage as backups in case a passage needed to be restarted. For this, 100 µl of cell suspension were added to 100 µl of 50% glycerol in 500-µl microtubes and stored in a −80°C freezer.

#### EcN uptake

EcN bacteria were fed individually to 14-day-old larvae according to an oral exposure protocol modified from Milutinović *et al.* [48]. The standardized overnight liquid bacterial cell solutions of the ancestral and the 10 passaged lines were therefore mixed with flour and pipetted to half of a 96-well plate per line with a volume of 20 µl/well. With a concentration of 5.3 × 10^10^ cells/g flour, each of the resulting flour discs contained about 1.6 × 10^8^ cells in total. The discs were left to dry overnight at 30°C. Per replicate line, 48 14-day-old larvae were exposed to the bacteria upon transferring larvae into these 96-well plates with the flour discs containing EcN. After 24 h of continuous exposure to EcN at 30°C and 70% humidity, individual larvae were transferred to 96-well plates containing flour discs without EcN. We thereby avoided direct transfer of EcN and extracted only bacteria that had persisted in the host, as described in the following sections.

#### Re-isolation of EcN

The bacteria were extracted at extending time points to maintain a selection pressure towards persistence in the host. In passages 1–4, EcN was extracted after 48 h being on the discs without EcN, in passages 5–7 after 72 h, and in passage 8 after 96 h.

Before the extraction of bacteria, all larvae were cleaned and surface sterilized with three steps. First by dipping into sterile water for 10 s to wash off residual flour, followed by dipping for 10 s into 70% ethanol, and dipping into sterile water for 10 s to remove residual ethanol. Three pools of 10 surface-sterilized larvae per line were transferred to 500-µl microtubes containing 200 µl PBS and a sterile metal bead. The larvae were homogenized using a Mixer Mill MM301 (Retsch) for 2 min at 28 Hz. The homogenates were then plated out on LB agar plates dyed with Congo Red and Coomassie Blue to allow the identification of red EcN colonies, which incorporate the dyes in their biofilm. After 24 h of incubation at 30°C, one colony per line was used to inoculate a new 50 ml overnight culture starting the next passage.

#### Flour disc passage

To test for a potential adaptation of EcN to the flour environment, six flour-passaged lines were treated similarly as the larvae-passaged lines but passaged only through flour discs (at 30°C and 70% humidity) that did not contain any larvae. In parallel to the extraction of EcN from larvae in the larvae-passaged lines, the flour discs with the flour-passaged lines were resuspended in 200 µl of PBS and streaked out on LB agar plates dyed with Congo Red and Coomassie Blue, using an inoculation loop. A single colony per control line was used to inoculate the liquid culture for the next passage.

### Persistence analysis

To analyze the persistence of the serially passaged EcN in the *T.castaneum* host, an infection experiment was performed that included all larvae-passaged EcN lines after eight passages, as well as the ancestral strain. For this, per EcN line, 144 larvae (i.e. 1584 larvae in total) were orally exposed to vegetative bacterial cells for 48 h. In contrast to the preceding passages, in which exposed larvae were sampled in pools of 10 larvae per passage cycle, we monitored persistence in detail, i.e. in daily intervals by checking the presence of EcN in 20 individual larvae per day and line. Droplets of 10 µl of 10 individual homogenates were applied per LB + Congo Red/Coomassie Blue plate. After overnight incubation at 30°C, the plates were screened for the presence or absence of EcN colonies.

### Growth dynamics

Possible differences in the growth dynamics of the passaged replicates were assessed by monitoring the turbidity of liquid cultures over time under aerobic and anaerobic conditions at different temperatures. Liquid LB medium cultures with a volume of 5 ml were inoculated with six larvae passaged replicates (L2, L3, L5, L6, L8, L9), six flour passaged replicates (F1–6), and six pseudoreplicates of the ancestral strain. Pseudoreplicates of the ancestral strain were produced by inoculating six clones in six different cultures. The samples were incubated overnight at 30°C and 180 rpm. All cultures were diluted to a relative absorbance of 0.5 at a wavelength of 600 nm (OD_600_). Of the standardized cell solutions, 10 µl were used to inoculate 200 µl of liquid LB medium (diluted 1:10 with PBS) in a 96-well plate. For each bacterial line, four technical replicates were measured every 15 min for 24 h at 30°C and 32°C under aerobic conditions and at 34°C under anaerobic conditions using the Infinite^®^ 200 PRO plate reader (Tecan).

### Motility analysis

Differences in the motility of bacterial lines can, similar to growth, indicate trade-offs linked to a putative adaptation to the novel larval environment. Differences in motility were assessed by measuring swarming distance on soft agar plates. The swimming motility of three technical replicates of six biological replicates per passage treatment (L2, L3, L5, L6, L8, L9, F1–6) and six pseudo-replicates of the ancestral strain was tested. Out of the 10 larvae-passaged lines, we chose 6 that showed a trend of elevated persistence. LB agar plates with 0.3% agar can be used to evaluate the motility of bacterial strains [61]. Bacterial cells were transferred from single colonies to the center of an LB 0.3% agar plate by slightly touching the agar surface, using the tip of a sterile toothpick. The plates were incubated at 30°C and the diameter of the swimming area was measured after 6 and 9 h.

To assess the flagella expression in the lines, the flagellin protein (FliC) was extracted from six replicates per treatment: ancestral (A1–6, pseudo-replicates), larvae-passaged (L2, L3, L5, L6, L8, L9) and flour-passaged (F1–6). Laboratory stock *E.coli* Nissle 1917 was used as a positive control and *E.coli* Nissle 1917 ΔfliC was used as a negative control; 15 ml cell cultures of all replicate lines were grown in LB medium. The flagella were sheared off and separated from the cells by vortexing and subsequent centrifugation. Before separating the remaining proteins by SDS-PAGE, the protein was denatured at 65°C. After blotting the gel, the protein was visualized using FliC antibodies.

### Colony morphology

To investigate possible differences in colony morphology, we grew three colonies of the following replicate lines on dye-supplemented agar plates: larvae-passaged (L2, L3, L5, L6, L8, L9), flour-passaged (F1–6) and ancestral (A1–6, pseudo-replicates). The synthesis of cellulose and curli fimbriae, components of the extracellular matrix were visualized using the dyes Calcofluor White (under UV) and Congo Red, respectively [62, 63]. While *E.coli* K-12 colonies generally produce more curli fimbriae than cellulose at 30°C, thus appearing as brown and smooth colonies, EcN displays a pdar (pink, dry and rough) colony morphotype [62, 64]. To test the colony morphology and the formation of an extracellular matrix, bacterial strains were streaked out on agar plates with added Congo Red or Calcoflour white and incubated at 30 and 37°C for 96 h.

### Whole-genome sequencing analysis

#### DNA extraction and whole-genome sequencing

The genomes of the three passaged lines L2, F4 and L9 were sequenced using next-generation sequencing. While L2 and F4 were chosen based on a trend for elevated persistence, L9 was put forward for sequencing because of its peculiar colony morphology. The total genomic DNA was isolated using the MagAttract^®^ HMW DNA kit (Qiagen, Hilden, Germany). To prepare 500 bp paired-end libraries of all isolates, we used the Nextera XT DNA Library Preparation kit (Illumina, San Diego, CA, USA). Libraries were sequenced on the Illumina MiSeq sequencing platform using v2 sequencing chemistry.

#### Quality control and variant calling

The quality of the raw sequencing data was analyzed using FastQC v0.11.5 [65]. Raw reads were trimmed using Sickle v1.33 [66]. Quality trimmed reads were then aligned against the in-house reference EcN genome (CP058217) using Burrows–Wheeler Aligner v0.7.17 [67]. The produced alignment was sorted, and duplicates were marked using Picard v2.17.3 (http://broadinstitute.github.io/picard/). Variant calling and filtering were performed using the Genome Analysis Toolkit (GATK) v3.8.0 [68]. The effects of the resulting variants were then annotated using SnpEff v4.3 [69].

#### De novo assembly and pan-genome analysis

Genome assembly of the processed reads was carried out with SPAdes v3.13.1 [70]. The assembled genomes were then annotated using Prokka v1.12 [71] and used for a pan-genome analysis, which was performed using Roary v3.12.0 [72]. The resulting presence–absence matrix of orthologous genes was visualized using FriPan [73].

### Effects of EcN on host mortality and gene expression

#### Oral exposure with EcN and Btt

To investigate the putative protective effect of EcN, larvae were pre-treated with the passaged EcN strains L2, L3 and L6 as well as with the ancestral strain (5.3 × 10^10^ cells/g flour) and subsequently exposed to *Btt* spores (3.3 × 10^10^ spores/g flour). Mortality was screened for 14 days and pupation/eclosure for 28 days. Dead larvae were identified by immobility, the characteristic body shape and a darkened color.

EcN cells were fed to 14-day-old larvae according to the modified oral exposure (‘pretreatment’) previously described (‘EcN uptake’). After 3 days on the EcN diet, the larvae were transferred to the *Btt* diet (‘treatment’). As additional controls, the commensal *E.coli* K-12 strain MG1655 (K-12) served as a general control for bacterial pretreatment, and unexposed PBS controls were included for both pretreatment and treatment.

The diet for oral exposure with *Btt* was made as described in Milutinović *et al.* [48]. In short, *Btt* from the frozen stock was plated on an LB agar plate and incubated overnight. The following day, 5 ml of BT medium [w/V–0.75% Bacto Peptone (Sigma), 0.1% glucose, 0.34% KH_2_PO_4_, 0.435% K_2_HPO_4_] was supplemented with 25 µl of sterile salt solution (0.2 M MgSO_4_, 2 mM MnSO_4_, 17 mM ZnSO_4_, 26 mM FeSO_4_) and 6.25 µl of sterile 1 M CaCl_2_ × 2H_2_O solution, inoculated with five single *Btt* colonies from the LB agar plate and incubated overnight in culture tubes (Simport) at 200 rpm. The following morning, 300 ml of BT medium were supplemented with 1.5 ml of salt solution, 375 µl 1M CaCl_2_ × 2H_2_O and inoculated with 5 ml of the overnight culture. The culture was incubated in a 2-l Erlenmeyer flask for 7 days at 180 rpm. After 7 days of sporulation, the spores were centrifuged at 4500 rpm for 15 min at RT, followed by washing with PBS and centrifuged again. *Btt* spores were counted using a Thoma counting chamber. The adjusted concentration of liquid bacterial cultures was mixed with flour and pipetted into 96-well plates. After drying the plates, larvae (previously exposed to EcN) were individually transferred to each well and monitored for survival and development.

#### Gene expression upon oral uptake of EcN

To characterize a possible differential immune reaction of *T.castaneum* to the exposure to EcN, the expression levels of several defense genes were assessed. These genes are either involved in the regulation of AMP expression or code for AMPs themselves. After oral exposure to EcN, *E.coli* K-12 MG1655 (K-12), or a PBS negative control for 3 days, 19 larvae per treatment were snap frozen in individual microtubes using liquid nitrogen. For the quantitative reverse transcription PCR (RT-qPCR), we used seven genes, including two housekeeping genes Rp49 and Rpl13a as in Ref. [74]. We used six replicates per bacterial treatment.

#### Quantitative reverse transcription PCR

Total RNA from six samples of five pooled frozen larvae for each treatment (EcN, K-12, PBS) was extracted using a combined protocol [74] of TriFast™ (VWR) and spin columns of the SV Total RNA Isolation System (Promega). The quantity and quality of extracted RNA were evaluated using a NanoPhotometer^®^ P-300 (Implen). Per sample 300 ng of RNA were used for cDNA synthesis with the RevertAid First Strand cDNA Synthesis Kit (Thermo Scientific). The reverse transcription was performed according to the manual of the kit. The RT-qPCR was run on a LightCycler^®^ 480 (Roche^®^) with SYBR^®^ Green fluorescent dye (Thermo Fischer Scientific). The relative expression of the AMPs Attacin2 (Att2), Cecropin2 (Cec2), Defensin2 (Def2), Defensin3 (Def3), and an Osiris16-like protein (Table 1 and Supplementary Data) was assessed using the housekeeping genes of the ribosomal protein L13a (Rpl13a), ribosomal protein 49 (Rp49) and control treatments [75]. Technical duplicates were set up for each sample.

**Table 1.** Sequences of primers used for RT-qPCR

Protein name	Forward primer sequence (5′–3′)	Reverse primer sequence (5′–3′)	Source
Att2	CAAACGACCAAAGGGAAACTAAA	TGAACTTGTCCAGTTGCATCGA	Yokoi *et al.* [96]
Cec2	GCCGAAGGAGCTGGAAGATTA	TGGTGGTGGAGGTTGTTGGTA	Yokoi *et al.* [96]
Def2	CCCTTTTCTGCATCTTCGAAAC	CACATGCGGAATGGTTTAGCT	Yokoi *et al*. [96]
Def3	TGCAATCACTGCTTACCCACTT	ACAAGCAGCATGATTCACTTTGA	Yokoi *et al.* [96]
Rp49	TTATGGCAAACTCAAACGCAAC	GGTAGCATGTGCTTCGTTTTG	Eggert *et al.* [74]
Rpl13a	GGCCGCAAGTTCTGTCAC	GGTGAATGGAGCCACTTGTT	Eggert *et al.* [74]
Osiris 16	CGACAAGCCTACTCCC	TGTAGTCGTCCTCCTCGTTC	Lindeza and Barth, 2020 unpublished data

### Statistics

All statistical analyses and figures were performed and produced in R [76] using RStudio [77]. For producing the plots, the packages ggplot2 [78] and ggpubr [79] were used.

To test for a normal distribution of the analyzed data, a Shapiro–Wilk test was performed [80]. Based on the normality test, a parametric or non-parametric test was conducted to assess significance.

Persistence data were analyzed using a binomial generalized linear model [81, 82] using the package ‘lme4’ [81].

Growth curves were analyzed using the package growthcurver [83].

The calculation of growth parameters bases on the following equation:

$$N_{t}\frac{K}{1+\left(\frac{K-N_{0}}{N_{0}}\right)e^{-rt}}.$$

Furthermore, we analyzed growth rate (*r*) and carrying capacity (*K*) with a Kruskal–Walllis test [84] followed by the non-parametric Wilcoxon signed-rank test [85] corrected for multiple testing using the Benjamini–Hochberg procedure [86].

Since the bacterial motility and AMP gene data and datasets were normally distributed, and assumptions were met, one-way ANOVA was performed. The means were compared using Tukey Honest Significant Differences [87, 88].

For the analysis of survival, a Cox Proportional Hazards Model was applied with one random effect [89–91] using coxph function from the ‘survival’ package [92, 93]. The treatment was defined as the fixed factor, while a putative plate effect was defined as a random factor. The assumptions were met and after fitting the model, the variance between treatments was assessed using a one-way analysis of variance. The means were compared using Tukey's test for *posthoc* analysis with Benjamini–Hochberg adjusted *P* values [94, 95].

## RESULTS

### Persistence of EcN in *T.castaneum* did not increase after serial passages

In the serial passage experiment, we expected to select for persistence in the beetle larvae. We thus focused on measuring the persistence of the passaged lines compared to the ancestral strain after completing eight infection cycles. The persistence of the passaged lines was compared to the ancestral strain by testing individual larvae for the presence of EcN.

**Figure 1.**
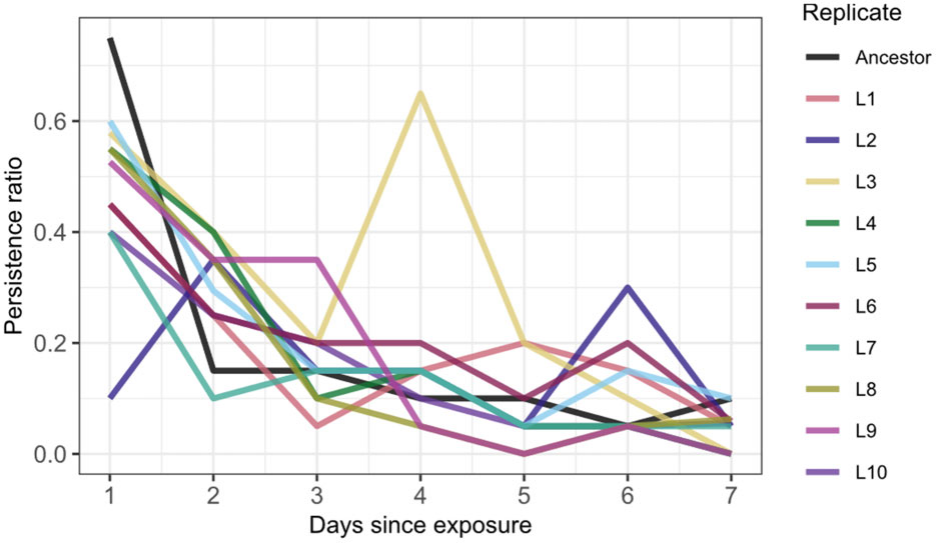
Persistence of EcN in *T.castaneum* larvae after serial passage. Proportion of larvae harboring EcN after 48 h of exposure. Ten passaged EcN lines (L1–10) and the ancestral strain were monitored. A total of 20 larvae per replicate and day were tested for bacterial presence or absence (*n* = 1584)

We did not detect a significant difference in the ability to persist in the beetle larvae between the ancestral strain and any of the passaged lines (Fig. 1). One day after exposure, the passaged EcN lines varied in persistence in the beetle larvae with an average persistence rate of 46%, while 75% of the tested larvae treated with the ancestral strain harbored EcN. Over time, the rate of larvae-harboring EcN decreased, and after 7 days, the mean persistence rate was 5%. In two lines (L9 and L10) EcN was not found anymore.

### Host and flour-passaged lines showed increased growth rate compared to ancestral EcN

**Figure 2.**
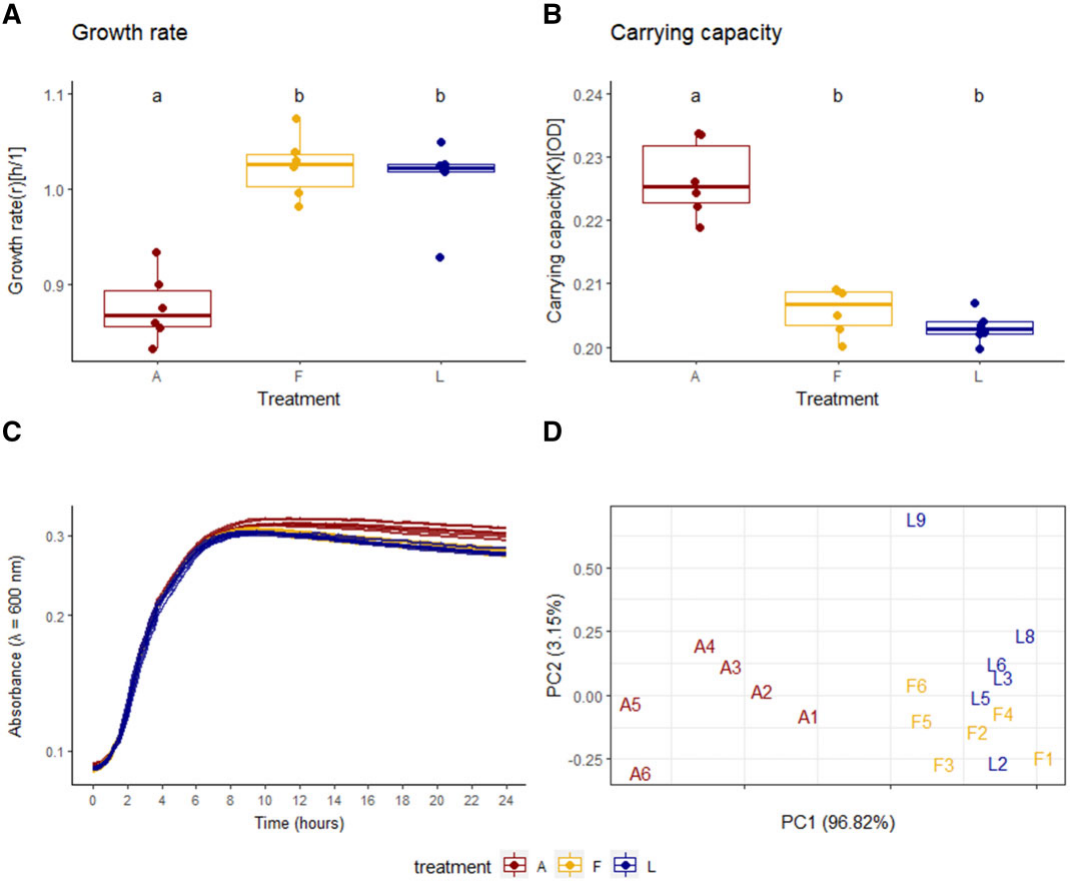
EcN growth curves and attributes. (**A**) Differences in the growth rates between the passaged lines and the ancestral strain (Kruskal–Wallis *X*^2^ = 10.889, Df = 2, *P* = 0.00432). (**B**) Differences in the carrying capacities (Kruskal–Wallis *X*^2^ = 12.316, Df = 2, *P* = 0.00211). (**C**) Optical density (absorbance) of bacterial liquid cultures at *λ* = 600 nm. (**D**) PCA of growth rate, carrying capacity and the area under the curve. Six replicates per treatment were analyzed: larvae-passaged (L2, L3, L5, L6, L8, L9), flour-passaged (F1–6), ancestral (A1–6, pseudo-replicates). Measurements were taken every 15 min for 24 h at 30°C. A, ancestral strain; F, flour-passaged bacteria; L, larvae-passaged bacteria. Statistical differences between the treatments are marked with letters a and b

To characterize putative trade-offs resulting from adaptation to the new environment upon serial passage, we investigated potential effects on bacterial growth parameters. The absorbance of liquid cultures inoculated with a standardized number of cells was monitored for 24 h at 30°C under aerobic conditions (Fig. 2).

The curves of all lines follow a similar, exponential growth (Fig. 2C). The maximum or intrinsic growth rate (*r*) turned out to be significantly higher in the evolved lines (Fig. 2A), while the ancestral lines appeared to grow to a higher density than the passaged lines. Analysis of the growth capacities (*K*) confirmed a significantly higher maximum density in the ancestral lines (Fig. 2B).

We performed a principal component analysis (PCA) of *r* and *K*, as well as the area under the curve to visualize similarities and differences in these attributes between the individual lines (Fig. 2D). The area under the curve is a conclusive parameter because it integrates the contributions of the growth rate, carrying capacity and area under the curve, into a single value. The PCA revealed that the growth parameters of the ancestral lines appear distinct from the evolved ones, which cluster together. The passaged line L9, however, showed distinctive growth characteristics dissimilar to all other lines (Fig. 2D).

However, the growth under aerobic conditions with an increased temperature of 32°C showed no significant difference between the lines. All of them follow a similar growth and there is no difference in growth rate and carrying capacity (Supplementary Fig. S3). The same was observed for growth under anaerobic conditions with strong variation among the replicate lines (Supplementary Fig. S4).

### Larvae- and flour-passaged EcN lines were more motile than the ancestral strain

After 6 h at 30°C, the larvae-passaged lines as well as the flour lines showed significantly higher motility than the ancestral strain (average swarming distance: L passaged lines: 4.76 mm, F passaged lines: 4.36 mm, A strain: 3.58 mm). This difference was still observed after 9 h of incubation. No significant difference between the passaged lines was detected but the larvae-passaged lines tended to be more motile than the flour-passaged lines at both time points. Despite the elevated motility of the passaged lines, the line L9 showed particularly low average swarming distances of 3.78 mm after 6 h and 8.06 mm after 9 h (Supplementary Fig. S5). However, no difference in flagella expression was detected between the lines (Supplementary Fig. S6).

### Colony morphology of one replicate line changed after passages through the host

One of the passaged lines, L9, appeared to display a slightly stronger fluorescence upon Calcofluor White staining, in addition to a more wrinkled colony outline, which may indicate increased cellulose expression (Fig. 4).

### Genome of EcN remained unchanged after serial passage

The morphology and the strongly diminished growth and motility of the host-passaged line L9 suggested that a mutation might have occurred and defined these properties. Therefore, we analyzed the genomic variability of the draft genome sequences of this and two other lines, which showed elevated motility (L2, F4). Both, variant detection and pan-genome analysis of these isolates showed the absence of genome-level differences compared to the ancestral strain.

### EcN did not provide a survival benefit to the host upon pathogen exposure

Beetle larvae were first orally exposed to EcN (‘pretreatment’), followed by oral exposure to *Btt* 3 days later (‘treatment’). EcN for the pretreatment was derived from the ancestral strain or either of three of the larvae-passaged EcN lines. For this, lines L2, L3 and L6 were selected, because they showed a tendency for above-average persistence (Fig. 1).

The *Btt* infection strongly reduced larval survival (Fig. 5), whereas the EcN pretreatment did not have any strong effect on larval survival of the *Btt* infection. Only larvae pretreated with one of the host-passaged EcN lines (L6) showed a slightly increased survival, while *E.coli* K-12 pretreatment slightly reduced survival, an effect that was significant only in direct comparison of these groups (*χ^2^* = 16.91, Df = 5, *P* = 0.0046) but not when compared to the PBS control.

In addition to assessing the effects of EcN exposure on survival, we monitored the pupation and eclosure rates of the larvae for 4 weeks but did not observe any significant differences (Supplementary Figs S7 and S8).

### Expression of immune-related genes did not change in *T.castaneum* upon oral uptake of EcN

We assessed the expression levels of several immune genes by RT-qPCR to characterize a possible differential immune reaction of *T.castaneum* to the exposure to ancestral EcN. The selected genes code for the AMPs Att2, Cec2, Def2 and Def2 [96], as well as for an Osiris16-like protein, which was found to be involved in oral immune priming with *Btt* [97]. The expression patterns were measured in larvae, after oral exposure to ancestral EcN or *E.coli* K-12 (as a control) for 72 h (six replicates of five pooled larvae per treatment). We did not find any statistically significant differences in gene expression to PBS-exposed control larvae (Fig. 6).

## DISCUSSION

Serial passage experiments are important tools to understand the evolution of bacteria in new environments, in particular new hosts, and to adapt bacteria to new experimental host systems. One of the main goals of this project was the establishment of a novel model system for studying the human probiotic EcN. An invertebrate system could facilitate research on this widely used probiotic.

We performed a serial passage experiment of the human probiotic bacterium EcN in an invertebrate host, larvae of the red flour beetle *T.castaneum*, as well as in their environment and food source, i.e. flour. We observed changes in some of the key parameters of the bacterial phenotype, which did not lead to increased persistence of the bacteria in this novel host, nor to any genetic changes.

Differences between vertebrate and invertebrate physiology are limitations that could prevent the attempted establishment of invertebrate model systems for vertebrate probiotics. Despite parallels between mammals and insects in how they deal with microorganisms, the immunological and physiological differences between the intestinal environments of *T.castaneum* and humans might prevent successful adaptation of *Ec*N to an invertebrate gut. One of the mechanisms important for *Ec*N colonization is binding to the host’s gastrointestinal mucus with the flagellum as the major adhesin [98]. Only recently, Dias *et al.* [99] showed that several insects produce mucous substances, including the mealworm beetle *Tenebrio molitor*, a close relative of *T.castaneum*. However, mammals and insects differ strongly in chemical and enzymatic composition of the gastrointestinal mucus layer [99, 100]. Moreover, the differing body temperature of the host species might be another important factor preventing adhesion and growth in the host gut [101, 102].

By extracting *Ec*N after an increasingly long duration in the larval system, selection for persistence was expected, but the selection pressure exerted by the novel environment might not have been sufficiently strong. A probable reason is an insufficient number of passages, given the reported low intrinsic mutation rates of *E.coli*: 4.1 × 10^−4^ for strain REL606 [103] to 1.0 × 10^−3^ mutations per genome per generation for K-12 strain MG1655 [104]. Estimating the number of generations that the bacterial lines went through in the performed serial passage experiment is rather difficult since it is not apparent if and how fast EcN replicates within the host or the flour. Jerome *et al.* [105] showed that adaptation of a human intestinal bacterium to a novel mouse model host is possible within as few as three passages but suggest that a fundamental ability to colonize a broad host spectrum and high standing genetic diversity may be crucial to long-term persistence. We started our serial passages with a single clone eliminating standing genetic diversity, which selection could have acted on. In addition, we propagated a single clone per line from one passage to the following. Thereby, we wanted to avoid clonal competition in the liquid culture phase, which bears the risk to counter-act adaptation that might have happened in the insect host, in particular if such adaptations come at the cost of reduced growth in liquid medium. The amplification of bacterial numbers in liquid culture was indispensable for infection of the next host generation. However, our approach relies on selection being strong enough in the host to produce genetic differences in the clone derived from the host phase. We might have lost potentially host-adapted genotypes that were still at too low frequency. Our procedure also creates bottlenecks that might have favored genetic drift. Additionally, the selection pressure exerted by desiccation in the flour might outweigh the effects of the serial passage in the host.

Both larvae and flour-passaged lines showed increased growth rates in liquid culture at 30°C under aerobic conditions, but a decreased carrying capacity relative to the ancestral strain (Fig. 2). Somerville *et al*. [106] also found significantly elevated growth rates in *Staphylococcus aureus* upon serial passage *in vitro* and suggested that the difference was based on the more efficient utilization of available nutrients upon serial passage. However, when the temperature was changed to 32°C or when growth was measured under anaerobic conditions, no difference in growth was observed. The loss of the already weak phenotypic differences under slightly differing conditions leads to the conclusion that the observed differences in growth are neither a stable nor a strong phenotype. This also corresponds to the lack of any genetic changes.

**Figure 3.**
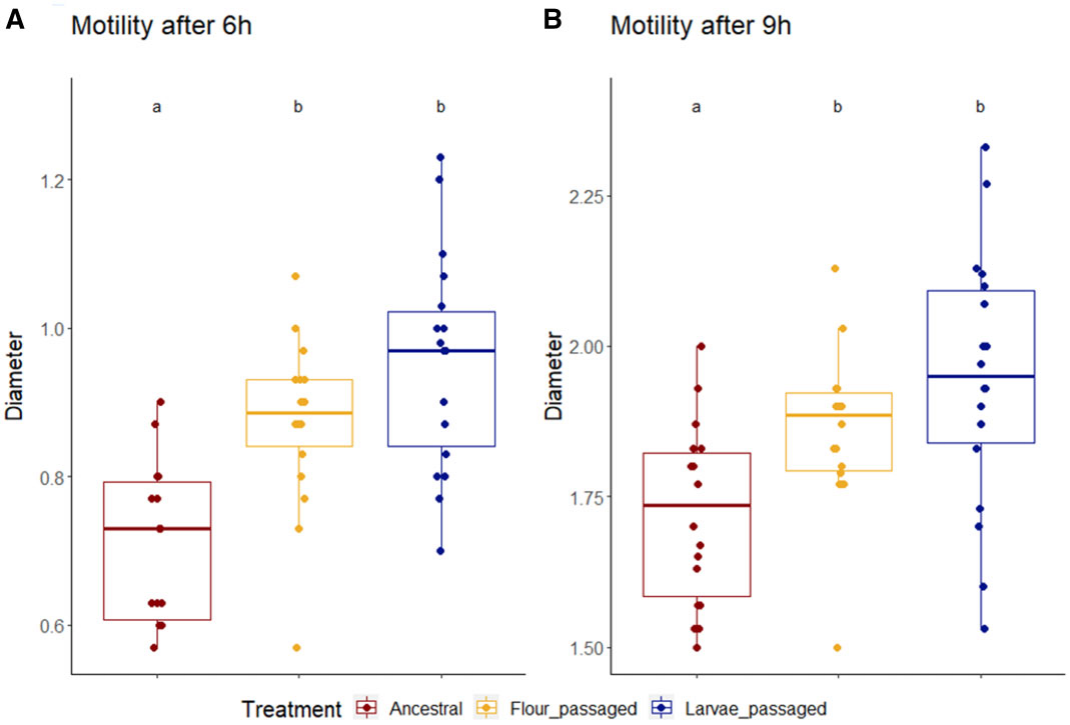
EcN motility. Differences in swarming ability between the evolved lines and the ancestral strain. The radius of the swarmed areas on 0.3% agar plates was measured after 6 h (3 A) and 9 h (3 B) post inoculation. Triplicates of six replicates per treatment were analyzed: larvae-passaged (L2, L3, L5, L6, L8, L9), flour-passaged (F1–6), ancestral (A1–6, pseudo-replicates). (**A**) Motility per treatment after 6 h (ANOVA, Df = 2, *F* = 17.4, *P* < 0.001). (**B**) Flagella motility after 9 h per treatment (ANOVA, Df = 2, *F* = 8.06, *P* < 0.001). Statistical differences between the treatments are marked with letters a and b

The assessment of motility upon passage showed a significantly higher swimming capability on soft agar for the evolved lines compared to the ancestral strain (Fig. 3). Taxis of *E.coli* bases on the expression of flagella and is thought to be very costly, leading to infrequent expression [107]. The biosynthesis of flagella has been shown to be strongly regulated by environmental factors including pH, salinity, temperature and presence of d-glucose [108, 109]. Moreover, Landini and Zehnder demonstrated that the flagellar motility of *E.coli* is induced by oxygen-limited conditions [110]. Thus, the difference in motility between the passaged lines and the ancestral strain might have resulted from the extreme environmental conditions during the passage, which could have promoted the expression of flagella. However, no significant difference in FliC expression was found, suggesting that an increased expression of flagellin might not be responsible for the observed difference in motility. The effect of variations in the amount of *E. coli* flagella on the cells’ swimming behavior has been found to be very limited [111]. Instead, differences in metabolism or intracellular signaling might have had an effect on the rotation frequency, changes of direction or the tumbling behavior of *E.coli* cells [112].

**Figure 4.**
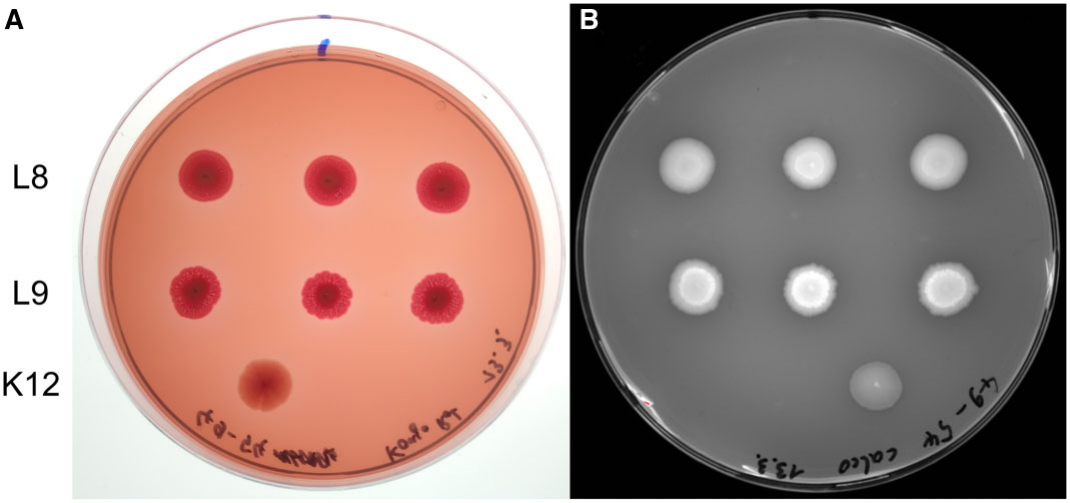
EcN L9 colony morphology. EcN colonies grown on Congo Red (left) and Calcofluor White plates (right). Triplicates of six replicates per treatment were tested: larvae-passaged (L2, L3, L5, L6, L8, L9), flour-passaged (F1–6), ancestral (A1–6, pseudo-replicates). Triplicates of the lines L8 (top row) and L9 (middle row) are shown. *Escherichia coli* K-12 MG1655 served as a control (bottom). The plates were grown for 96 h at 30°C. Morphology of the line L8 showed typical EcN morphology

The larvae-passaged line L9 appeared to display a slightly stronger fluorescence upon Calcofluor White staining, in addition to a more wrinkled colony outline (Fig. 4). An increased fluorescence on Calcofluor White suggests a higher cellulose concentration in the extracellular matrix, which is linked to the wrinkled colony phenotype [63]. In addition to its colony morphology, L9 displays differences in growth and does not cluster with the other larvae-passaged lines in the PCA on growth parameters (Fig. 2). Since L9 is one of the lines that were passaged through the host, it is conceivable, that the novel phenotype arose in an adaptive process to the larval environment. However, the low persistence of L9 indicates that this phenotype is not associated with colonization success (Fig. 1).

Nevertheless, the peculiar features of L9 are a proof of principle that serial passage of EcN can generate differential phenotypic characteristics. The morphology and the strongly diminished growth and motility of the larvae-passaged line L9 suggested that a mutation might have occurred and defined these properties. Despite the strong phenotypic changes that line L9 shows after serial passage through the host, we do not know how stable these changes are, since they were not tested after culturing without selection in the host or flour. Epigenetic mechanisms such as inheritance of DNA methylation patterns are recognized in bacterial biology [113]. Considering we did not observe any genomic differences, we suggest that the observed phenotypic differences could arise from epigenetic processes, but further analyses are needed to confirm this.

**Figure 5.**
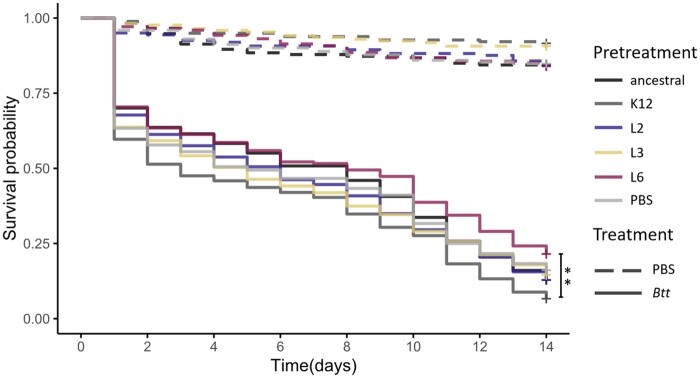
*Tribolium castaneum* survival upon EcN pretreatment. Survival of 14-day-old beetle larvae exposed to EcN-containing flour diet for 72 h (5.3 × 10^10^ cells/g flour), before transfer to *Btt*-containing flour (3.3 × 10^10^ spores/g flour). Three passaged EcN strains (L2, L3, L6) as well as the ancestral strain were used for pretreatment. PBS and K-12 strain MG1655 served as a negative control for pretreatment and treatment. The larvae were individualized in 96-well plates (*n* = 2128). **P = 0.0046. Full lines show survival of larvae challenged with *Btt* while dashed lines are PBS control

Oral previous exposure of *T.castaneum* larvae to serially passaged or ancestral EcN, prior to exposure to the entomopathogen *Btt*, did not significantly improve host survival (Fig. 5). Only one of the larvae-passaged lines (L6) appears to induce a slightly elevated survival rate, compared to pretreatment with *E.coli* K-12 strain MG1655. In conclusion, EcN does not seem to act as a probiotic in *T.castaneum* that protects against pathogens, at least not against *Btt*.

The antagonistic activity of EcN against pathogens might need specific requirements and an unaccustomed microbiome, as well as suboptimal environmental conditions in the beetle gut might have impaired its probiotic effect in the novel host. For example, iron homeostasis has been shown to play a major role in infectious diseases and successfully competing for iron is thought to be a central mechanism of the antagonistic effect of EcN against pathogens [14, 114]. Furthermore, Sassone-Corsi *et al.* [13] demonstrated that elevated concentrations of environmental iron inhibit the microcin production of EcN. Moreover, the bactericidal activity of EcN’s siderophore microcins M and H47 has been shown to be restricted to members of the *Enterobacteriaceae* family with no activity against gram-positive bacteria, including *Bacillus* [12].

Another probiotic effect of EcN could be the enhancement of the integrity of the gut epithelium. In mammals, EcN induces the upregulation of the structural protein Zonula occludens-1 (ZO*-*1), which binds to both, actin filaments and the tight junction protein occludin, structurally linking tight junctions to the cytoskeleton [26, 27, 115]. The insect homolog of ZO-1 is the *Drosophila* discs-large tumor suppressor protein (Dgl), which has been shown to have a similar function to that of ZO-1 in septate junctions, the invertebrate homolog of tight junctions [116, 117]. ZO-1 and Dgl, however, show distinct differences in the secondary and tertiary protein structure and the regulation of expression might differ considerably despite the homology in function [116]. These dissimilarities could adversely affect or completely impair the stabilizing effect of EcN on the epithelial permeability of *T.castaneum*.

**Figure 6.**
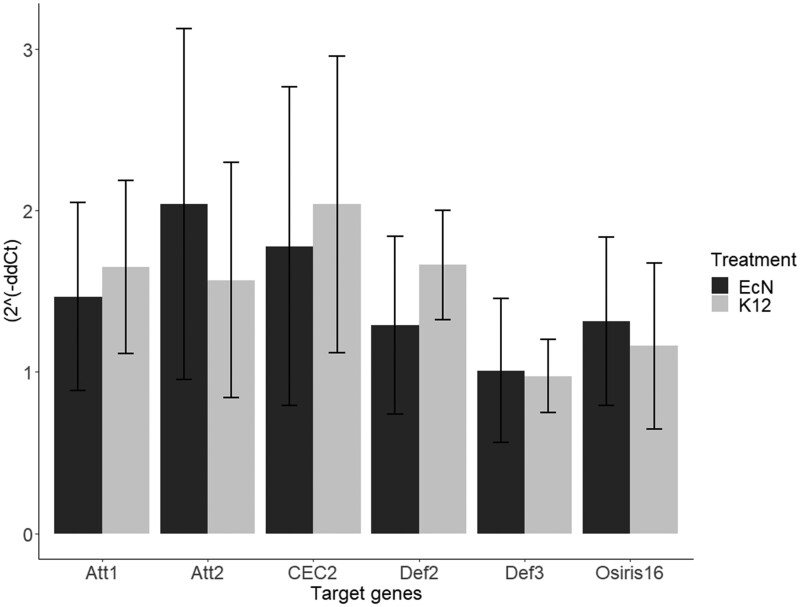
Differential expression of AMPs and Osiris16. Expression patterns assessed by RT-qPCR on RNA extracted from six replicates of five pooled larvae/treatment. *Tribolium castaneum* larvae that were orally exposed to EcN, *E. coli* K-12 MG1655 and PBS for 72 h. The genes coding for the AMPs Att2, Cec2, Def2 and Def3 as well as for an Osiris16-like protein were analyzed. ΔΔCp values were calculated using the expression of the housekeeping genes ribosomal protein L13a (Rpl13a), ribosomal protein 49 (Rp49) and PBS treatment as negative control

The absence of any effects on survival, pupation and eclosure of beetles after EcN exposure as well as expression levels of several tested immune genes (mostly AMPs; Fig. 6) suggest that there was no impact on the immune response and development of *T.castaneum.* Yokoi *et al.* [96] investigated the expression of AMPs upon exposure to multiple microorganisms including *E.coli* strain DH5α and found all of the AMPs that were tested in the present study upregulated. However, the effect was observed in pupae and the microorganisms used in their study were directly injected, instead of oral exposure [96]. However, similar immune genes as tested here (Osiris16-like) were differentially regulated upon oral infection or priming of *T.castaneum* with the entomopathogen *Btt* [97, 118], pointing towards a less immunogenic effect of oral exposure to EcN compared to *Btt*.

## CONCLUSION AND IMPLICATIONS

In conclusion, this exploratory study did not show any increased colonization success or protective effect of EcN after eight serial passages through *T.castaneum* larvae. However, we observed phenotypic changes in some growth attributes and motility in lines that were passaged through the host as well as those passaged through flour only. Moreover, the peculiar characteristics of one of the lines (L9) show that serial passage of EcN can generate differential phenotypes, even in the absence of changes on the genomic level. A longer duration of the serial passage experiment might be necessary to observe stronger phenotypic and potentially also genomic changes and yield further valuable insight into the adaptation of EcN to a novel host environment. The lack of robust alternative phenotypes indicates that EcN is rather stable in the environment. If this could be confirmed under further environmental conditions, it bears relevance regarding the safety of this widely used probiotic.

Studies such as ours on genome plasticity of EcN in the context of intestinal colonization or persistence in different host models improve the safety associated with the use of EcN as a probiotic by increasing our understanding of possible selective conditions in the host as well as bacterial genes and phenotypes under selection pressure. By understanding further details of the ecological fitness and genomic stability of EcN in different host environments, the safe therapeutic use of this probiotic can be improved.

## AUTHOR CONTRIBUTIONS

Conceptualization: N.C.H.S., A.K., J.K., investigation: N.C.H.S., A.K., O.M., data analysis: A.K., N.C.H.S., H.W., writing the original draft: N.C.H.S, A.K., editing and writing: N.C.H.S., A.K., H.W, J.K., U.D., funding acquisition: J.K and U.D.

## SUPPLEMENTARY DATA

Supplementary data is available at *EMPH* online.

## Supplementary Material

eoac001_Supplementary_Data
