# Supplementary material for: Serial passage in an insect host indicates genetic stability of the human probiotic Escherichia coli Nissle 1917
Source: Evol Med Public Health. 2022 Feb 11;10(1):71–86. doi: 10.1093/emph/eoac001 (PMC8853844; doi:10.1093/emph/eoac001)
Supplement: eoac001_Supplementary_Data [file eoac001_supplementary_data.docx]

**Serial passage in an insect host indicates genetic stability of the human probiotic *E. coli* Nissle 1917**

Nicolas C. H. Schröder, Ana Korša, Haleluya Wami, Olena Mantel, Ulrich Dobrindt, Joachim Kurtz

# Supplemental information


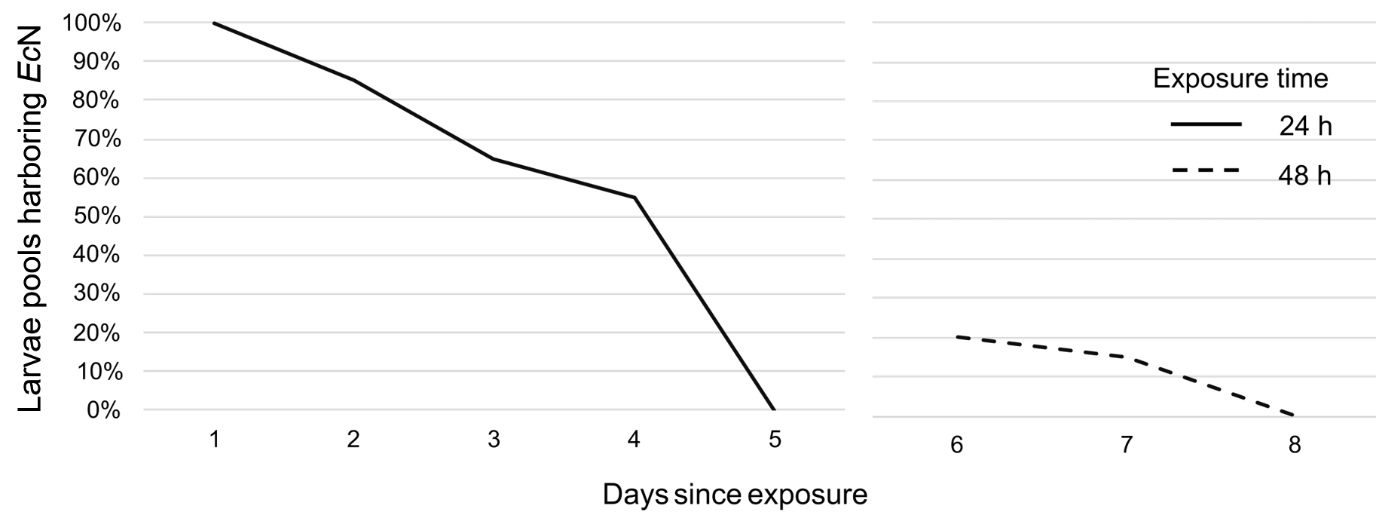


**Figure S1: Persistence of *Ec*N in *T. castaneum* larvae.** The proportion of larvae harboring *Ec*N after 24 h and 48 h of oral exposure. Per day 20 pools of 10 larvae were tested for bacterial abundance (n = 160).


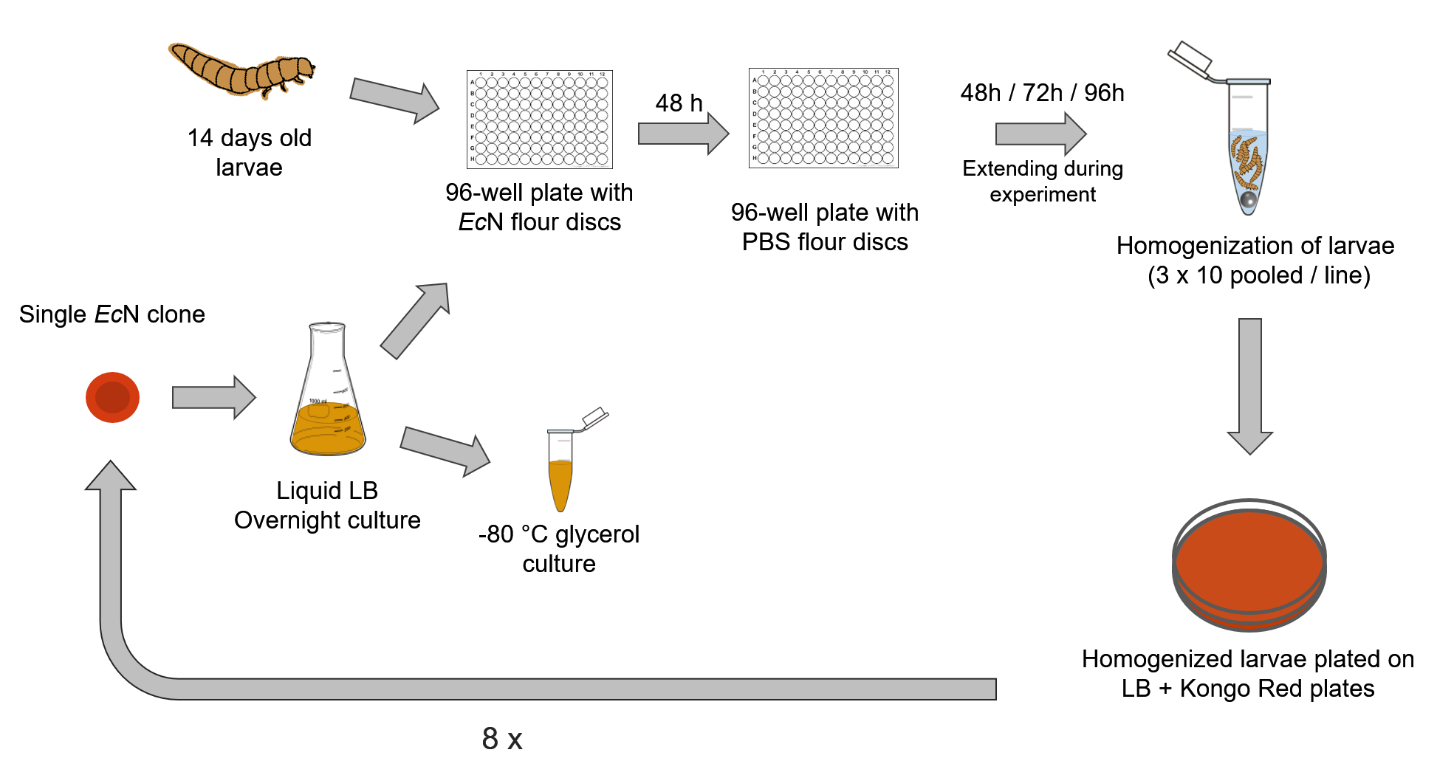


**Figure S2: Serial passage workflow:** 48 larvae were used per replicate line per passage. 10 replicate lines were passaged through the beetle larvae. In addition, 6 replicate lines were treated similarly, but were exposed to the flour environment (without beetle larvae) only.


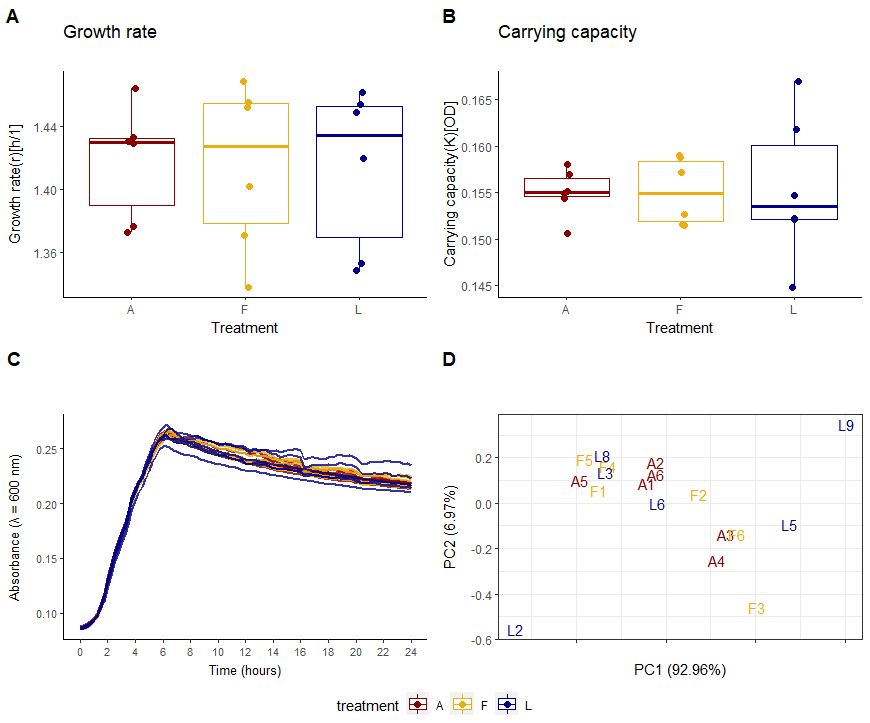


**Figure S3: EcN growth curves and attributes at 32°C.** A) Growth rates (Kruskal-Wallis *X^2^* = 0.03, Df = 2, p = 0.98). B) Carrying capacity (Kruskal-Wallis *X^2^* = 0.01, Df = 2, p = 0.99). C) Optical density (absorbance) of bacterial liquid cultures at λ = 600 nm. D) Principal component analysis of growth rate, carrying capacity and the area under the curve. Six replicates per treatment were analyzed: Larvae-passaged (L2, L3, L5, L6, L8, L9), flour-passaged (F1-6), ancestral (A1-6, pseudo-replicates). Measurements were taken every 15 min for 24 h at 32 °C. A - ancestral strain, F - flour-passaged bacteria, L - larvae-passaged bacteria.


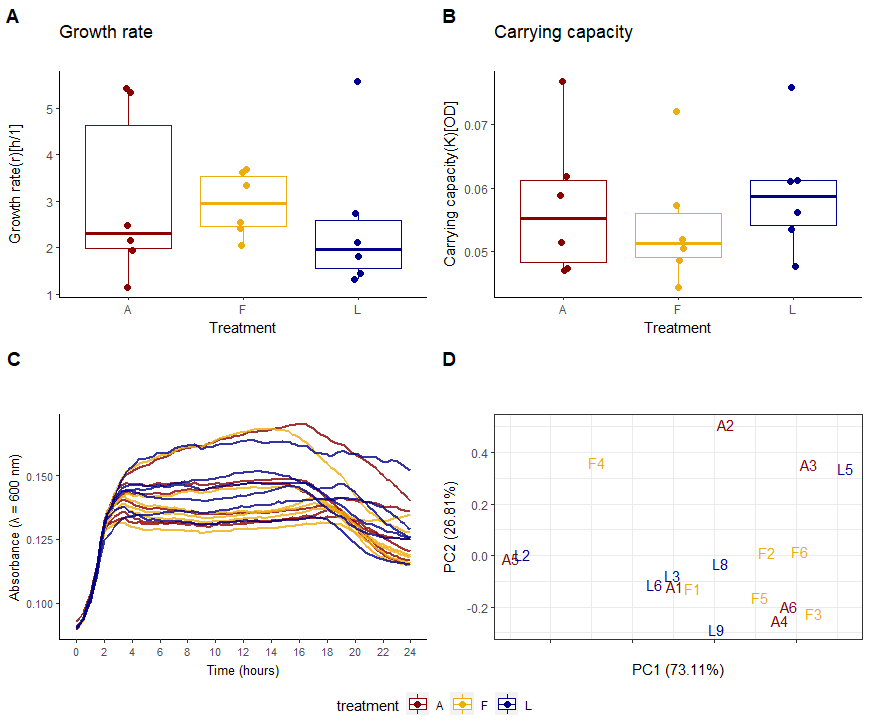


**Figure S4: EcN growth curves and attributes at 34°C in anaerobic conditions.** A) Growth rates (Kruskal-Wallis *X^2^* = 1.41, Df = 2, p = 0.49). B) Carrying capacity (Kruskal-Wallis *X^2^* = 1.16, Df = 2, p = 0.55). C) Optical density (absorbance) of bacterial liquid cultures at λ = 600 nm. D) Principal component analysis of growth rate, carrying capacity and the area under the curve. Six replicates per treatment were analyzed: Larvae-passaged (L2, L3, L5, L6, L8, L9), flour-passaged (F1-6), ancestral (A1-6, pseudo-replicates). Measurements were taken every 15 min for 24 h at 30 °C. A - ancestral strain, F - flour-passaged bacteria, L - larvae-passaged bacteria.


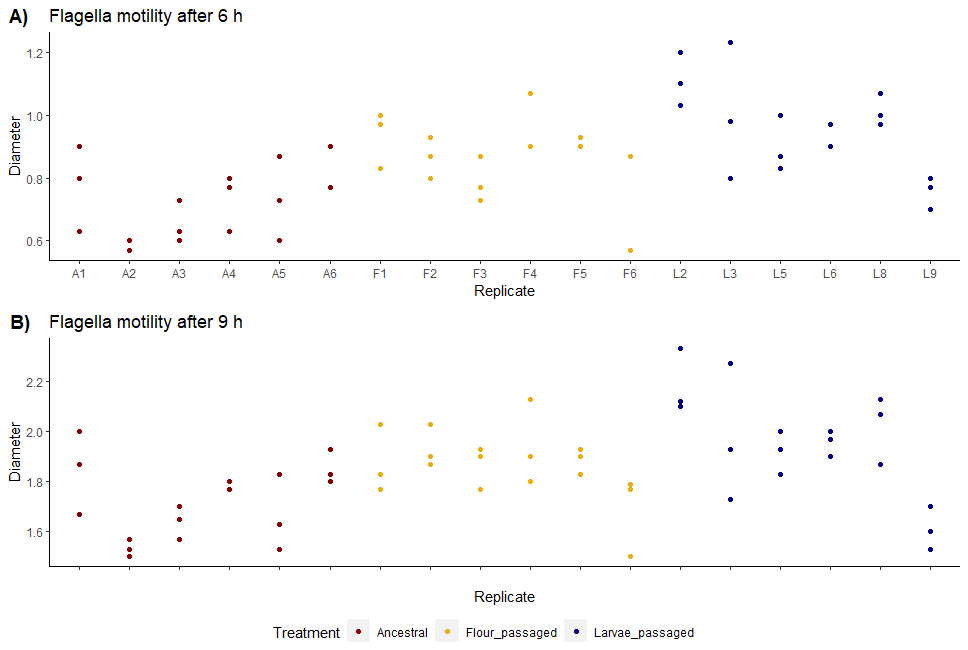


**Figure S5: Differences in swarming ability between the individual replicates of the evolved lines and the ancestral strain.** Triplicates of six replicates per treatment were analyzed. The radius of the swarmed areas on 0.3 % agar plates was measured after 6 h (A) and 9 h (3B) post inoculation.


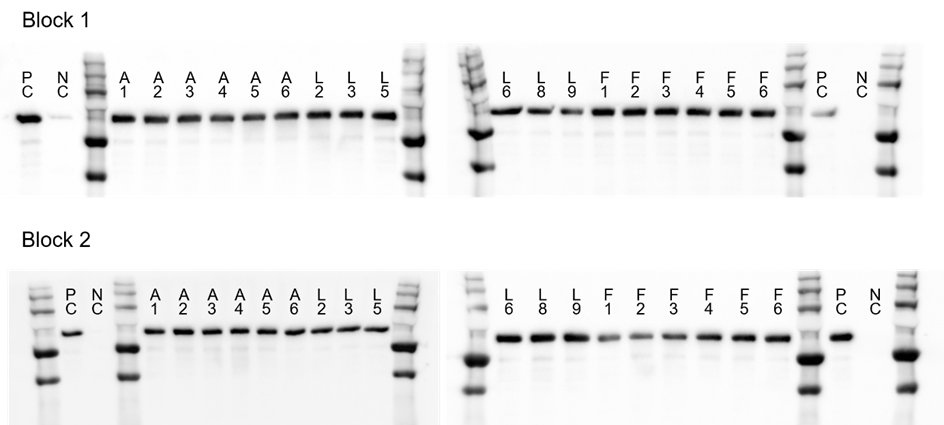


**Figure S6: Western Blot of FliC extraction.** Six replicates per treatment were analyzed: Ancestral (A1-6, pseudo-replicates), larvae-passaged (L2, L3, L5, L6, L8, L9) and flour-passaged (F1-6). Laborartory stock *E. coli* Nissle 1917 was used as a positive control (PC) and *E. coli* Nissle 1917 ΔfliC was used as a negative control (NC). The flagella were shered off and separated from the cells by vortexing. After blotting, the protein was visualized using FliC antibodies.


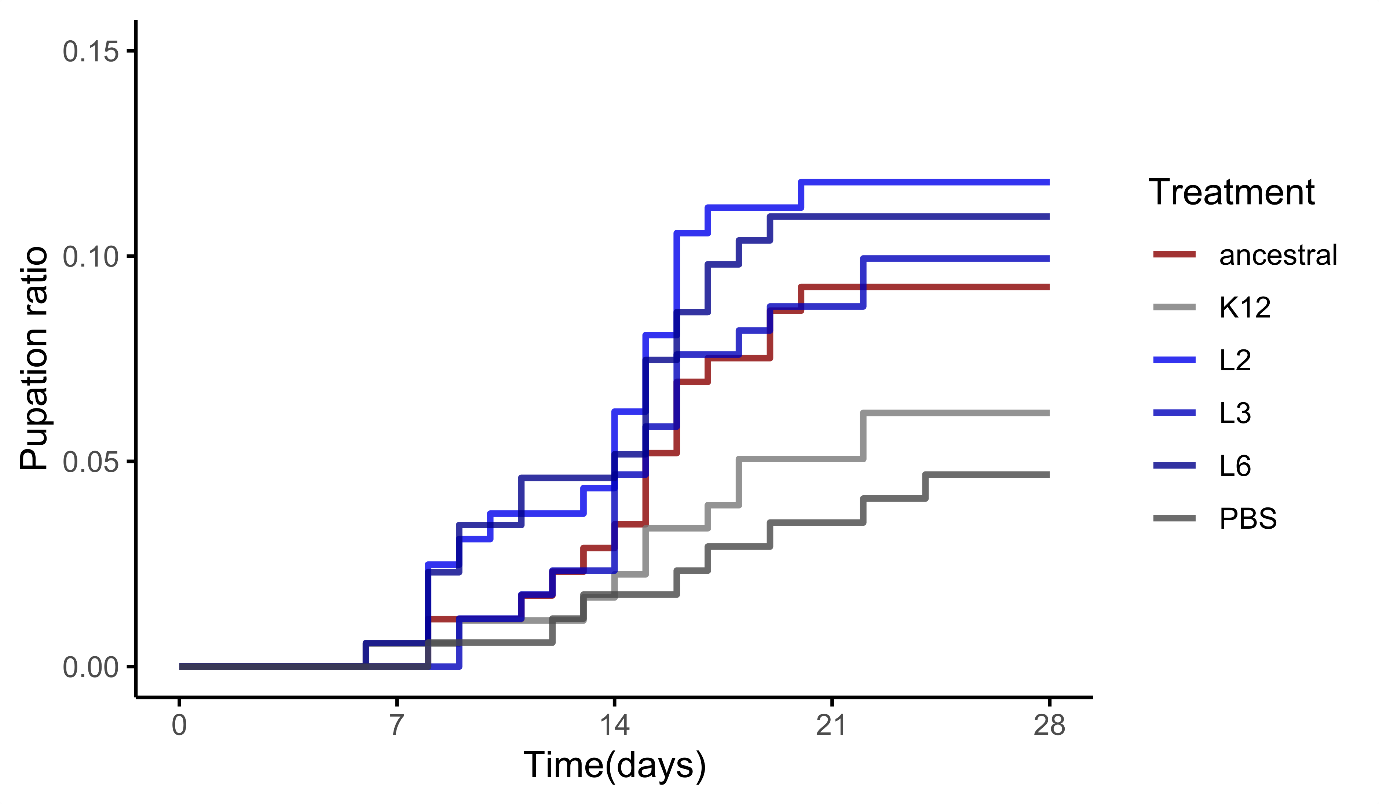


**Figure S7: *T. castaneum* pupation upon *E. coli* treatment.** The proportion of pupated beetle larvae (14 days old) exposed to *E. coli*-containing flour diet for 72 h (5.3 x 1010 cells / g flour), before transfer to PBS. Three passaged *Ec*N strains (L2, L3, L6), as well as the ancestral strain, were used for pretreatment. PBS served as a negative control for pretreatment and treatment. The larvae were individualized in 96-well plates (n = 1028).


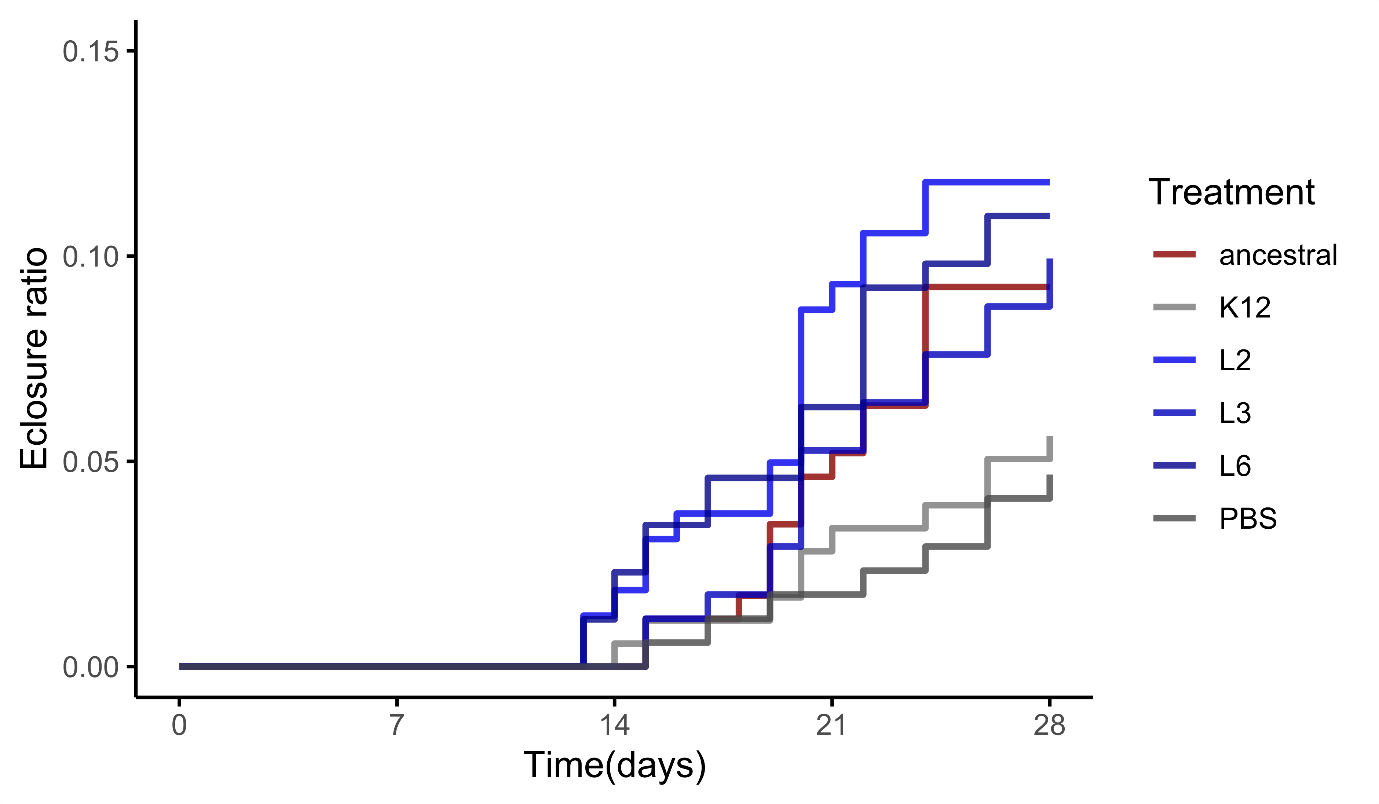


**Figure S8: *T. castaneum* eclosure upon *E. coli* treatment.** The proportion of beetle larvae (14 days old) exposed to *E. coli*-containing flour diet for 72 h (5.3 x 1010 cells / g flour), before transfer to PBS. Three passaged *Ec*N strains (L2, L3, L6), as well as the ancestral strain, were used for pretreatment. PBS served as a negative control for pretreatment and treatment. The larvae were individualized in 96-well plates (n = 1028).

Table 1. Sequences of primers used for RT- qPCR

| Protein name | Forward primer sequence (5′–3′) | Reverse primer sequence (5′–3′) | Source |
| --- | --- | --- | --- |
| Att2 | CAAACGACCAAAGGGAAACTAAA | TGAACTTGTCCAGTTGCATCGA | Yokoi et al., 2012) |
| Cec2 | GCCGAAGGAGCTGGAAGATTA | TGGTGGTGGAGGTTGTTGGTA | Yokoi et al., 2012 |
| Def2 | CCCTTTTCTGCATCTTCGAAAC | CACATGCGGAATGGTTTAGCT | Yokoi et al., 2012 |
| Def3 | TGCAATCACTGCTTACCCACTT | ACAAGCAGCATGATTCACTTTGA | Yokoi et al., 2012 |
| Rp49 | TTATGGCAAACTCAAACGCAAC | GGTAGCATGTGCTTCGTTTTG | Eggert et al.,2014 |
| Rpl13a | GGCCGCAAGTTCTGTCAC | GGTGAATGGAGCCACTTGTT | Eggert et.al, 2014 |
| Osiris 16 | CGACAAGCCTACTCCC | TGTAGTCGTCCTCCTCGTTC | Lindeza and Barth, 2020 unpublished data. |
